# Simultaneous Determination of Antibodies to Pertussis Toxin and Adenylate Cyclase Toxin Improves Serological Diagnosis of Pertussis

**DOI:** 10.3390/diagnostics11020180

**Published:** 2021-01-27

**Authors:** Aapo Knuutila, Alex-Mikael Barkoff, Jussi Mertsola, Radim Osicka, Peter Sebo, Qiushui He

**Affiliations:** 1 Institute of Biomedicine, University of Turku, Kiinamyllynkatu 10, 20520 Turku, Finland; aajukn@utu.fi (A.K.); ambark@utu.fi (A.-M.B.); 2 Department of Pediatrics and Adolescent Medicine, Turku University Hospital, Kiinamyllynkatu 4–8, 20520 Turku, Finland; jusmer@utu.fi; 3 Laboratory of Molecular Biology of Bacterial Pathogens, Institute of Microbiology of the Czech Academy of Sciences, Videnska 1083, 142 20 Prague, Czech Republic; osicka@biomed.cas.cz (R.O.); sebo@biomed.cas.cz (P.S.); 4 Department of Medical Microbiology, Capital Medical University, No. 10 Xi Tou Tiao, You’an Men Wai, Feng Tai District, Beijing 100069, China; 5 Research Center for Infections and Immunity, Institute of Biomedicine, University of Turku, 20520 Turku, Finland

**Keywords:** Pertussis, in vitro diagnostics, point-of-care, serology, lateral flow, multiplex

## Abstract

Serological diagnosis of pertussis is mainly based on anti-pertussis toxin (PT) IgG antibodies. Since PT is included in all acellular vaccines (ACV), serological assays do not differentiate antibodies induced by ACVs and infection. Adenylate cyclase toxin (ACT) is not included in the ACVs, which makes it a promising candidate for pertussis serology with the specific aim of separating infection- and ACV-induced antibodies. A multiplex lateral flow test with PT and ACT antigens was developed to measure serum antibodies from pertussis-seropositive patients (*n* = 46), healthy controls (*n* = 102), and subjects who received a booster dose of ACV containing PT, filamentous hemagglutinin, and pertactin (*n* = 67) with paired sera collected before and one month after the vaccination. If the diagnosis was solely based on anti-PT antibodies, 98.5–44.8% specificity (before and after vaccination, respectively) and 78.2% sensitivity were achieved, whereas if ACT was used in combination with PT, the sensitivity of the assay increased to 91.3% without compromising specificity. No increase in the level of anti-ACT antibodies was found after vaccination. This exploratory study indicates that the use of ACT for serology would be beneficial in combination with a lower quantitative cutoff for anti-PT antibodies, and particularly in children and adolescents who frequently receive booster vaccinations.

## 1. Introduction

*Bordetella pertussis* continuously circulates in the population [1]. Pertussis toxin (PT) is a unique antigen for *B. pertussis,* and therefore the recommended antigen for serological analysis of infections. However, PT-based serology suffers one important drawback: extensive use of acellular pertussis vaccines that deliver substantial amounts of PT complicates the serological diagnosis by elevating anti-PT IgG levels in vaccinated individuals for several years [2,3,4]. The same issue applies for other *B. pertussis* antigens pertactin (PRN), filamentous hemagglutinin (FHA), and fimbriae. Additionally, other *Bordetellae* also induces cross-reacting antibodies to these antigens, other than PT. To improve the serological diagnosis of pertussis, antigens not included in the currently used acellular vaccines should be considered for differentiation of recent infection from vaccination. The adenylate cyclase toxin (ACT), involved in the suppression of host immunity in the early phases of colonization, is a well-characterized antigen that induces respectable antibody responses during infection [5,6,7,8,9,10]. A combinatory antibody test with well-established quantitative cutoff values for anti-PT antibodies to demonstrate specificity [11], and with ACT to differentiate between infection and recent vaccination, could improve pertussis diagnostics [9,10]. We earlier reported a quantitative and rapid lateral flow (LF) platform, based on immunochromatography, for multiplex determination of antibody response to PT, PRN, and FHA antigens without the complexity of common laboratory practicalities [12]. The developed multiplex LF platform was further used in this study to measure anti-PT and anti-ACT antibody responses from patients, healthy controls, and acellular pertussis vaccine recipients.

## 2. Materials and Methods

### 2.1. Serum Samples and Reference Assays

A total of 282 samples were included in the study (Table 1). Sixty-seven subjects were included from Finnish adolescents who received a booster dose of a dtap vaccine containing PT, FHA, and PRN (Boostrix, GlaxoSmithKline, Rixensart, Belgium), and paired serum samples were collected before and one month after vaccination [13]. Serum IgG antibodies to PT of these samples had been previously measured by ELISA. One hundred and two serum samples, collected in 2016 for a Finnish seroprevalence study from 20–29-year-old Finns, with undetectable IgG anti-PT antibody result (≤1 IU/mL), were included as a control group. Forty-six Finnish patients with respiratory symptoms and serologically diagnosed pertussis (2015–2016) were selected based on a positive combination of IgA and IgM antibody levels measured by ELISA using sonicated *B. pertussis* bacteria as a coating antigen, and with the criteria of anti-PT IgG concentrations higher than 50 international units (IU)/mL [14]. 

Anti-PT IgG antibodies of control and patient samples were measured with standardized ELISA at the Finnish National Reference Laboratory for Pertussis as previously described [15,16]. The CyaA-AC^−^ toxoid [17] used as ACT antigen and the soluble AC domain antigen were produced and purified as described [18,19]. Anti-ACT IgG levels were measured by a similar simplified ELISA assay for all samples: in short, wells (Nunc 96-well plate, catalogue No. 269,620, ThermoFisher Scientific, Roskilde, Denmark) were coated overnight in RT with 0.2 µg/mL of ACT in 50 mM sodium bicarbonate buffer (pH 9.6). Wells were blocked with 1% bovine serum albumin-PBS (BSA, art. 810,033, MP Biomedicals, USA), and serum samples were added in 1:60 dilution in 1% BSA-PBS. WHO standard 06/142 (NIBSC, PottersBar, UK) was used as a positive control. Although there is no official reference serum for anti-ACT antibodies available, this standard produced a respectable signal response both in ELISA (an average absorbance of 0.9–1.1) and LF assays (35,000–39,000 fluorescence counts). Absorbance at 405 nm was measured with Multiskan EX device (Thermo Scientific, Vantaa, Finland) from an alkaline phosphatase secondary antibody-mediated reaction after ten minutes.

### 2.2. Lateral Flow Test Strips and Multiplex Assay

**Figure 1:**
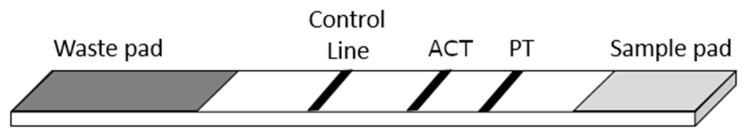
The layout of the multiplex lateral flow test. Pertussis toxin (PT) and adenylate cyclase toxin (ACT) antigen test lines were absorbed on the nitrocellulose membrane, in respective order.

The preparation of the strips and the test procedure were done similar to Knuutila et al. with the following modifications [12]: 1000 ng/cm of ACT was used as the second test line, between native PT (GlaxoSmithKline Biologicals, S.A., Rixensart, Belgium) and the control line (Figure 1 and Figure S1). 06/142 sera, in-house PT-positive and PT-negative controls were included for each run. Duplicate LF test strips of each sample were measured after an overnight drying in RT, covered from light. The 102 anti-PT IgG negative samples were tested with test strips containing only the ACT test line and control line.

### 2.3. Statistics

Data were analyzed using IBM SPSS statistics 27.0 software for Windows (IBM Corp., Armonk, NY, USA). The differences in means between the groups were tested with Mann–Whitney *U*-tests with Bonferroni corrections, and two-sided *p*-values less than 0.05 were considered as statistically significant. Paired *t*-tests were performed for comparison of means within the pre- and post-vaccination samples. Correlation of LF and ELISA results were calculated with the Spearman’s correlation coefficient.

## 3. Results

### 3.1. Specificity and Overall Assay Performance

**Figure 2:**
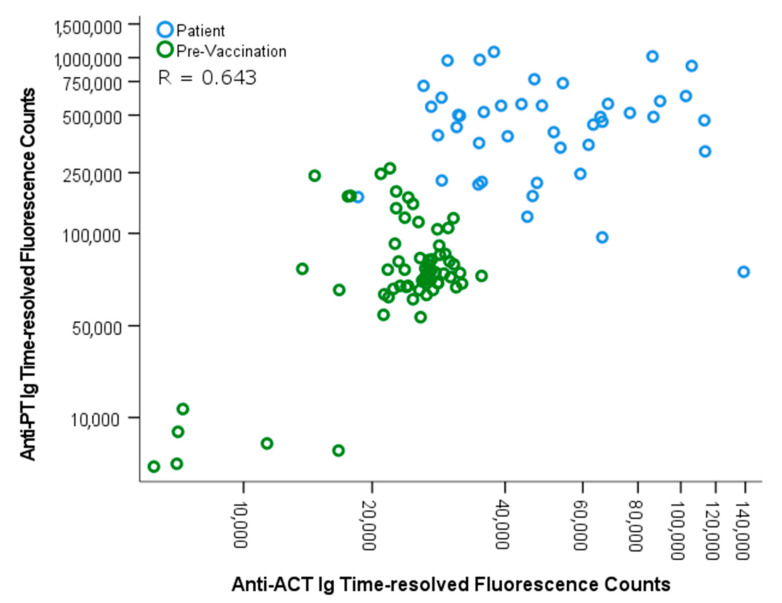
Spearman correlation between anti-PT and anti-ACT antibodies was evaluated with pertussis patients (*n* = 46) and pre-vaccination samples (*n* = 67) in the LF assay. One outlier from patients is limited out with over 300,000 ACT counts. LF: lateral flow.

A simple multiplex lateral flow assay was developed for the detection of anti-PT and anti-ACT antibodies from sera taken from individuals from four groups; PT-IgG seronegative, PT-IgG seropositive, and before and after pertussis vaccination backgrounds. The average coefficient of variation in the LF assay between two replicates was 6.99% for the ACT and 7.38% PT test line. Specificity was established based on the panel of healthy controls: for ACT, the average signal + 2 × standard deviation resulted in roughly below 40,000 counts; as for PT antibodies, we earlier reported a limit of quantification of 20 IU/mL with LF assays [20], corresponding to 48,000 counts in the assay. Among patients, the signal responses correlated well between ELISA and LF assays with a Spearman correlation of 0.698 for ACT, and 0.712 with PT, whereas among vaccination samples, ACT correlated only by a factor of 0.130 between the assays, and 0.835 with PT (Figure S2). Of note, ELISA results are for IgG antibodies, whereas protein A, which measures IgG, IgA, and IgM, was used in the LF assays. ACT and PT responses correlated moderately well among the patient and pre-vaccination samples (R = 0.643) (Figure 2), and post-vaccination samples understandably deviated from that trend.

**Figure 3:**
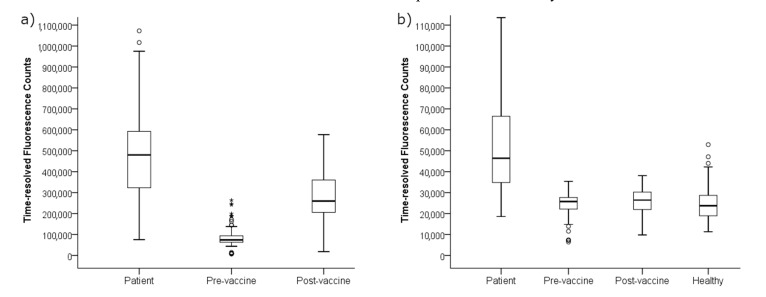
The multiplex antibody readouts from lateral flow test strips were measured as average time-resolved fluorescence signal from two test lines containing (**a**) PT and (**b**) ACT with pertussis patient samples (*n* = 46), paired before and one month after vaccination samples (*n* = 67) and with healthy controls (*n* = 102, only tested with ACT). Two outliers from patients are limited out with over 140,000 counts in Figure (**b**). A significant difference in anti-PT antibodies was noted between pre-vaccination antibodies and both the patients and post-vaccination antibodies (*p* < 0.001). Patients had significantly higher anti-ACT antibodies in comparison to other study groups (*p* < 0.001).

Pre- and post-vaccination samples demonstrated good specificity among the tested antigens. For PT, which is a part of the vaccine, signal increase by LF was on average 6.8-fold, whereas ACT signal levels did not change (average fold increase by 1.1) after vaccination (Figure 3). A clear increase of PT antibodies after vaccinations was observed in 65/67 of cases in LF, and 63/67 in ELISA (defined as an increase of 25% in fluorescence signals or international units/mL). Only one of the deviating samples was the same between assays. For ACT, the signal slightly increased in five of the paired samples and remained the same in 62 samples in the LF assay.

### 3.2. Differentiation between Vaccination and Infection 

Initially, 36/46 patient samples were considered positive by a cutoff of 100 IU/mL PT-IgG by ELISA. A similar positivity rate of 36/46 was accomplished by LF, corresponding to a signal threshold of 250,000 counts of the WHO standard. Out of the 10 patient samples which scored negative by this definition, six more cases scored positive based on a combinatory signal threshold of 110,000 signal counts (corresponding to 50 IU/mL) of PT-IgG and 40,000 counts of ACT, as defined in this study. Of the 41 vaccination samples between the 110,000 and 250,000 anti-PT signal range, all samples scored negative for anti-ACT antibodies (Figure 3 and Table 2). Thereafter, in total, the sensitivity of the assay increased to 91.3% from 78.3%, without compromising specificity. If ACT screening was applied for all samples exceeding 110,000 PT counts, none of the vaccination samples would remain false positive. However, the test would lose 30% more positive cases (*n* = 14), reaching 100% specificity and 60.9% sensitivity. Without ACT screening and by using solely the lower PT-signal level as diagnostic cutoff, the test would have ended with 97.8% sensitivity and 41.0% specificity.

## 4. Discussion

The currently recommended serological diagnosis of pertussis is based on assays that measure anti-PT IgG antibodies. These assays, however, do not differentiate antibodies induced by the acellular vaccine and by infection. To overcome this issue, multiple approaches have been considered to either measure immunoglobulin subclass, isotype, or different combinations of other pertussis-relevant antigens simultaneously [21,22,23,24,25]. The adenylate cyclase toxin produced by *B. pertussis* is not included in the acellular vaccines and induces infection specific antibodies, which makes it a promising candidate for pertussis serology with the specific aim of distinguishing vaccination and infection-induced antibody responses. So far in this regard, anti-ACT IgG in combination with anti-PT IgA was previously noted to improve pertussis serodiagnosis [9].

It is recommended that a cutoff of ≥100 IU/mL PT-IgG antibodies indicates a recent pertussis infection within a year (without taking a booster dose of dtap vaccine) and that ≥50 IU/mL indicates a recent infection in the past few years [11]. In this present study, serum anti-PT antibodies were first determined by ELISA in three groups of individuals including serologically confirmed patients, healthy controls, and vaccine recipients who received a booster dose of three-component acellular vaccine, and anti-ACT antibodies were then measured. A multiplex LF test with PT and ACT antigens was further used to measure these samples. When ACT was used in combination with PT, the sensitivity of the assay increased from 78.3% to 91.3%, indicating that the use of ACT would be especially beneficial in combination with a lower (50–100 IU/mL) quantitative cutoff for anti-PT antibodies (Table 2) [11]. Furthermore, after vaccination, a significant increase of anti-PT antibodies was observed as expected (*p* < 0.001), whereas anti-ACT antibodies remained unchanged. Overall, the levels of anti-ACT antibodies in patients were significantly higher in comparison to other study groups (*p* < 0.001). With all these observations in mind, the use of ACT could significantly improve both sensitivity and specificity of serodiagnosis. Antibodies to ACT are reportedly rather low after whole-cell vaccination, even as low as after the vaccination with acellular vaccines without ACT [26,27]. Of interest, the study by Cherry et al. showed that unvaccinated children with pertussis produced more antibodies to ACT than those children who were vaccinated (with either DTP or dtap) and were then later infected. This may lead to, in both whole-cell and acellular vaccinated populations, false-negative ACT results within infections. Certainly, more studies in this regard are needed.

Vaccination sera used in the study were collected one month after vaccination when antibody levels to vaccine antigens are substantially increased. Due to decreasing kinetics of antibodies [3,26], the test performance would likely be positively affected if samples after a longer period would be tested. On the other hand, a factor compromising the utility of the assay relates to ACT expression by other *Bordetellae*, and antibodies to common RTX motifs from other bacteria that may cross-react with ACT [5,27,28,29]. Thus, a domain of 400 amino acids of the N terminus of ACT specific only to ACT has been successfully used to avoid the complication caused by RTX motifs [10]. Also in our data, high anti-ACT antibodies were found in several of the individuals in the healthy cohort (*n* = 6/102) which would reflect this issue. We briefly evaluated the use of ACT and AC domain in the ELISA and LF assays, and the antigens correlated well within the patient samples (R = 0.879 and R = 0.710, respectively). None of the healthy samples tested by AC-domain LF exceeded the set 40,000 ACT signal cutoff (data is not shown), which would suggest a similar benefit for improved specificity. In the end, however, in terms of overall assay performance, the whole ACT antigen was more suitable and was therefore selected for the LF assay. In this aspect, future efforts should be focused on investigating the use of the AC domain, as it would likely even further improve the diagnostic distinction between healthy individuals and patients.

The study has certain limitations. The number of subjects for the patient group was limited, which consisted of persons within a wide range of age. Studies with a comprehensive amount of samples from all age groups paired with either a positive diagnosis by culture or PCR would be best suited. The sera selected for the acellular vaccination group have been stored for over 20 years. Although the sera have been stored without earlier thawing, the possibility and magnitude of degradation of antibodies are difficult to evaluate.

This study demonstrated that the multiplex measurement of PT and ACT antibodies can improve the serological diagnosis of pertussis. In combination with a rapid and easy-to-perform multiplex platform with lateral flow assays [12,20], serological testing could be performed very flexibly and even within a short period from pertussis vaccination. The test would be of particular use for serological diagnosis concerning children and adolescents who have recently received a booster vaccine, and in cases when the vaccination background of a patient is uncertain with regard to personal recalling or knowledge, or due to a lack of extensive records of the timing of the latest vaccination. Our preliminary results also warrant further studies in other populations since the circulation of cross-reacting bacteria may be different. Particularly, this study considered only populations in which acellular pertussis vaccines are being used, and anti-ACT antibody levels in those where whole-cell pertussis vaccines are in use may differ.

## Figures and Tables

**Table 1:** Study subjects.

	N	Age (Range)	Median ELISA Anti-PT IgG (IU/mL) (Range)
Patient	46	3–70	283 (50–1041)
Healthy	102	20–39	0–1
Pre-vaccination	67	11–13	9 (3–279)
Post-vaccination	67	11–13	109 (15–755)

**Table 2:** Positive results for individual and combinatory Ig antibody test using PT and ACT antigens in patient and vaccination study groups by LF assay.

Diagnostic Antigen(s)	Patients, Total *n* = 46 (Sensitivity %)	Pre-Vaccination, Total *n* = 67 (Specificity %)	Post-Vaccination, Total *n* = 67 (Specificity %)
PT ^1^	36 (78.2)	1 (98.5)	37 (44.8)
ACT ^2^	31 (69.6)	0 (100)	0 (100)
PT and ACT ^3^	42 (91.3)	1 (98.5)	37 (44.8)

^1^ Cases above 250,000 signal counts at the PT test line. ^2^ Cases above 40,000 signal counts at the ACT test line. ^3^ Cases above 250,000 signal counts at the PT test line OR cases between 110,000 and 250,000 signal counts at the PT test line and above 40,000 signal counts at the ACT test line.

## Data Availability

The data presented in this study are available on request from the corresponding author.

## Supplementary Materials

The following are available online at https://www.mdpi.com/2075-4418/11/2/180/s1, Figure S1: The multiplex readouts from lateral flow test strips, Figure S2: Correlation between ELISA and LF assays.
