# Supplementary material for: Simultaneous Determination of Antibodies to Pertussis Toxin and Adenylate Cyclase Toxin Improves Serological Diagnosis of Pertussis
Source: Diagnostics (Basel). 2021 Jan 27;11(2):180. doi: 10.3390/diagnostics11020180 (PMC7912298; doi:10.3390/diagnostics11020180)
Supplement: Supplementary file 1 [file diagnostics-11-00180-s001.pdf]

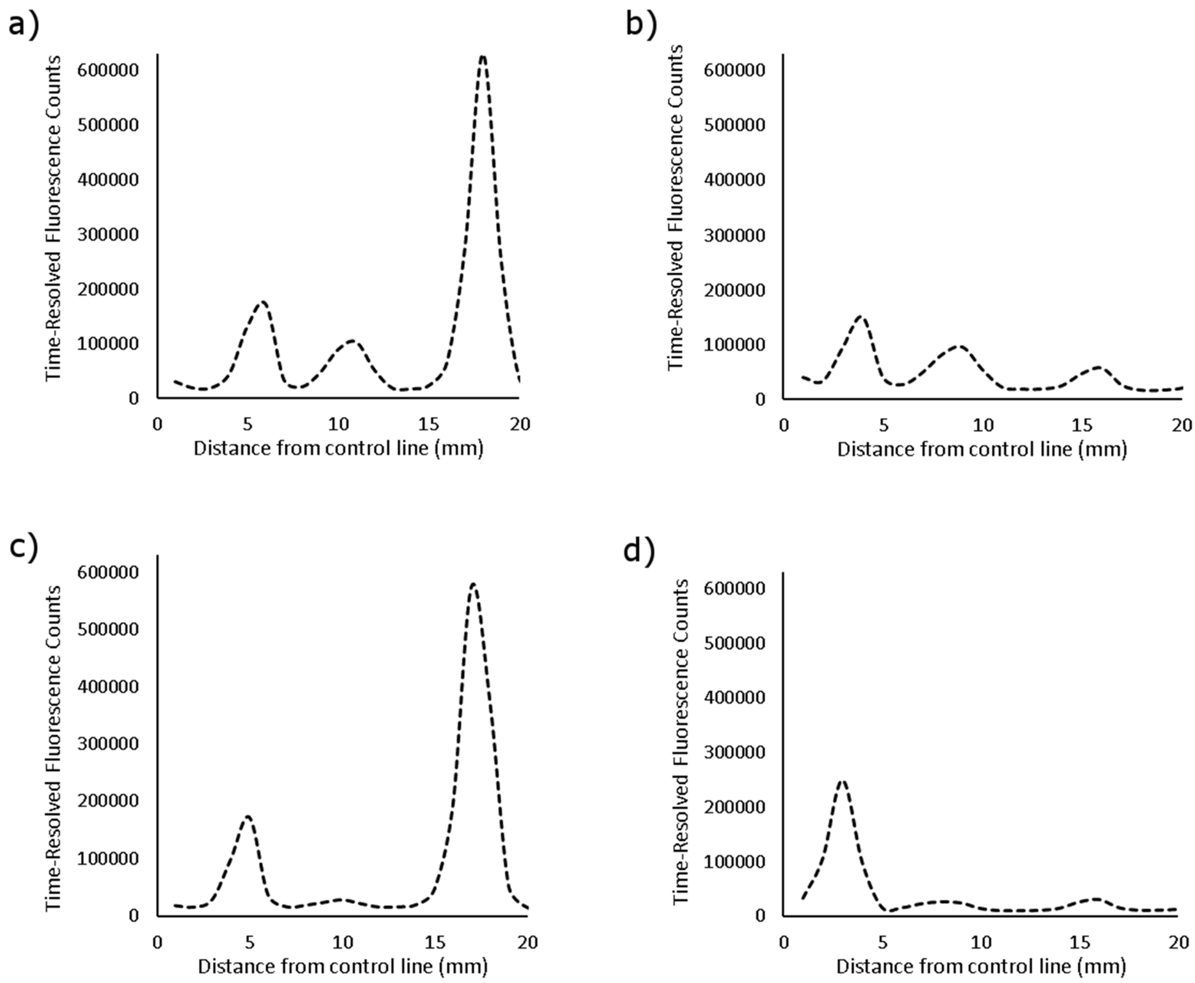

Supplementary Figure S1. The multiplex readouts from lateral flow test strips were measured as average time-resolved fluorescence signal by line scanning the nitrocellulose membrane starting from the control line (4 mm), followed by ACT (9 mm) and PT (17 mm) test lines. Examples of different result combinations included (**a**) ACT and PT Ig positive, (**b**) only ACT Ig positive, (**c**) only PT Ig positive, (**d**) ACT and PT Ig negative samples.

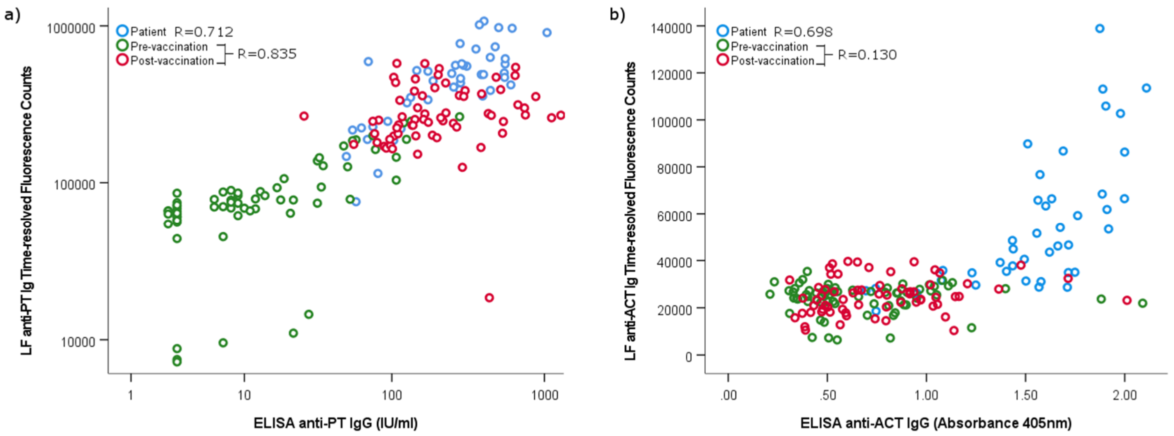

Supplementary Figure S2. Spearman correlation between ELISA and LF assays for (**a**) anti-PT antibodies and (**b**) anti-ACT antibodies were evaluated with pertussis patient and paired vaccination samples. One outlier from patients is limited out with over 300 000 fluorescence counts in figure (**b**).
